# Improving Research of Children Using a Rights-Based Approach: A Case Study of Some Psychological Research About Socioeconomic Status

**DOI:** 10.3389/fpsyg.2012.00293

**Published:** 2012-08-20

**Authors:** Tara M. Collins

**Affiliations:** ^1^Landon Pearson Resource Centre for the Study of Childhood and Children's Rights, Carleton UniversityOttawa, ON, Canada

## Introduction

Socioeconomic status (SES) and other social determinants of health are “identified as top priorities for action” in research [Canadian Coalition for the Rights of Children (CCRC), 2011, p. 42]. Consequently, SES is increasingly popular in some psychological and neuroscience research in order to expand upon understanding of its relationship to child development. In sum, this research demonstrates: “Growing up in a family with low SES is associated with substantially worse health and impaired psychological well-being, and impaired cognitive and emotional development throughout the lifespan” (Hackman et al., 2010, p. 651). Expanding the SES focus to include consideration of child rights (CR) in the research process, structure, and results would advance better understanding of children and improve research about them.

This brief article inquires: how would CR assist research about children by psychologists interested in neuroscience and SES? In short, children have human rights, which involve “the right to be properly researched” (Knowing Children, 2010). Indeed, Steinmetz (2010), p. 12 states that all our knowledge about how “abnormal child development” adversely affects the child's brain structure and capacity can be ultimately traced back to the disrespect of his/her rights. As such, CR should inform efforts related to researching the relationship between SES and neuroscience.

This brief commentary recognizes SES includes “occupations and thus the underlying levels of education and resulting incomes of the adult members of a household” (Johnson et al., 2007, p. 526). First, this article describes a child RBA (CRBA). Then, a CRBA frames analysis of some recent neuroscience and SES research and review articles before concluding.

## What is a CRBA?

Child rights are outlined in various legal instruments but essentially elaborated in the Convention on the Rights of the Child (CRC), adopted by the United Nations, (1989). The CRC has 193 States Parties (UN Treaty Collection, 2011), more than any other international rights treaty. It defines a child as anyone under 18 years and enunciates civil, political, economic, social, and cultural rights. The UN Committee on the Rights of the Child (1991, III) has identified four CRC provisions as general principles that inform implementation: non-discrimination, best interests, survival and development, and views of the child/participation. CR are fundamentally important and have revolutionized understanding about children.

Child rights influence all processes concerning children (Collins, 2008) and provide a framework in a CRBA to analyze populations and situations. A CRBA is important in (Collins, 2007) *inter alia* advancing respect, allowing measurement of progress, and improving effectiveness and sustainability of efforts (United Nations and OHCHR, 1989; Chinkin, 2001).

Yet, most researchers inadequately consider CR upon their efforts and results (Collins, 2007). While psychologists have made significant contributions to better understanding about children, room for progress remains in neuroscience research. For example, a recent review does not identify CRBA as a future direction (Hackman and Farah, 2009, p. 69).

## How Can CRBA Improve Research?

Child rights are relevant to research about children in at least two ways. First, CR highlight the importance and nature of the evidence base, which should be accurate without bias, reflect context, and respect the subjects. Second, as CR regulate relationships (Pearson, 2012) in terms of how children should be respected, researchers should consider their relationships to children they study. Researchers may be well-meaning in attempting to capture child needs, but efforts may not respect his/her rights. For example, researchers across disciplines commonly question: “What is a child?” (i.e., Schapiro, 1999; Stainton Rogers, 2003). From a rights-based perspective, this question should be reframed as: “who is a child?” to support understanding of the child as a subject, not an object (Van Bueren, 1998), and influence research and analysis.

In practice, a CRBA can be guided by the aforementioned CRC's four guiding principles (Collins, 2007) to support research and improve understanding of children. To illustrate, these principles now guide discussion about how a CRBA has and must influence neuroscience research.

## A CRBA to Research

### Non-discrimination

Convention on the Rights of the Child a.2 outlines CR shall not be hindered by discrimination on various grounds. Consequently, this principle demands consideration of every child and his/her group identity(ies) to prevent exclusion or marginalization in research (Collins, 2007) with appropriate data disaggregation to highlight discrimination.

Accordingly, some researchers recognize the diversity of children in their work but improvements are generally needed to advance non-discrimination. For instance, Lovejoy et al. (2000), p. 572 categorize all children in their meta-analysis about parenting into only three age categories, the final grouping of those “6 years and older” up to 16 years of age. This category blurs potential analytical distinctions about children who experience huge developmental changes over this significant age range. However, Lipina et al. (2005) and others reflect progress in fuller appreciation of children in relation to executive function, previously understood to be adult-only capacity, overcoming inaccurate, discriminatory assumptions about children. Lipina et al. (2005) also pay attention to the role of culture in their research, noting that children's skills, cognitive strategies, and brain organization may be influenced. These findings reflect the positive demands of non-discrimination principle in research practice and should be emulated. Spera (2005, p. 141) acknowledges context in parenting and that more investigations are needed about the “larger cultural and economic context in which families reside.” Such recognition of the complexities affecting children also reflects non-discrimination and is a valuable research direction.

Others also recognize discrimination's potential influence upon research and results including:

- Noble et al. (2012) who identify their results are not explained by gender, race, or IQ. Gender dimensions for instance, concern both girls and boys in terms of brain development and other influences (Younger et al., 2005).
- Johnson et al. (2007) who investigate the relationship between SES and school grades in biological and adoptive families.

While these studies are positive contributions, researchers can generally improve elaboration of, and respect of the non-discrimination principle.

The negative history of academic psychology, as Raizada and Kishiyama (2010), pp. 1–2, 8 describe, has involved researchers attempting to attribute learning difficulties “to genetic inferiority” and the stereotyping of people in poverty as “somehow inferior or undeserving.” However, material wealth does not always determine success since research found the 25% of Canadian children who are not school-ready includes middle-class [Canadian Coalition for the Rights of Children (CCRC), 2011, p. 52]. Further, if children are less successful on such scientists’ measures as IQ, and subject to labeling as “low SES” or having “deficits,” what are the implications for children and their development? Moreover, as attention to SES has sprung from some children's adverse development paths, then the negative starting point will inevitably affect the research questions, process, and results. It is important that more positive children's characteristics including resilience are identified (Hackman and Farah, 2009) and explored in research.

Power is a relevant issue in research as Esterberg outlines:

“…researchers need to address the power relationships… Researchers…often tend to be of a higher social class than the research participants…determine how the research is conducted…set the agenda and determine what is important…Research participants…do not typically have the power to determine, ultimately, how the data are used” (qtd. in Grover, 2004, p. 89).

Twelve-year-olds Maxine, John, and Stones confirmed: “Adults have power over children. Children aren't as respected” (Collins, unpublished). This power dynamic can result in discriminatory research. Thus, a CRBA to researching SES demands not only representation of different populations as study subjects, but also greater attention to how discrimination may affect children and research. The development of appropriate, standard categorizations would advance research to reflect greater diversity of children and respect non-discrimination.

### Best interests

The best interests of the child shall be “a primary consideration” in all actions concerning children according to CRC a.3, requiring a child focus. Neuroscience can improve its respect of this challenging principle since children are not always the explicit concern. For example, one paper identifies interest in neural data and behavior yet neglects to identify “child” in the paper's title or abstract (Raizada and Kishiyama, 2010). But some research reflects this principle well including Lipina et al. (2005) who concluded that executive function is no longer understood as only an adult capacity. To be clear, assumptions that ignore or exclude either the uniqueness and/or capacities of the child are problematic to best interests.

This principle is relevant to some psychology definitions of children's success, which do not necessarily reflect CR. While school readiness (Han et al., 2012), grades (Johnson et al., 2007), and other such achievement concerns are societally pertinent, should we only define children by such priorities, rather than for instance children's efforts or processes pertinent to brain development? The Roeher Institute (2002) recognizes children's different developmental paths are not reflected in much research resulting with: children's exclusion or interpretation as failures due to inability to achieve the measured outcomes; and/or negation of children with disabilities. Thus, critical analysis of research tools and methods is required to determine whether they exclude, rather than assess, children in accordance with best interests and a CRBA (Collins, 2008).

### Maximum survival and development

The child's right to maximum survival and development (CRC a.6) requires support of health, developing capacities, and abilities. Research should thus measure improvement or worsening of health and development over time (Collins, 2007). Accordingly, valuable findings from neuroimaging illustrate the impact on the developing brain of neglect and other stressors (Guyda et al., 2006) and support warm parental care during early childhood for brain maturation (Rao et al., 2010). Gianaros and Manuck (2010) acknowledge potential changes to SES at the individual and community levels over time will benefit future research. This is positive since the environment encircling the child influences his/her development as Bronfenbrenner, (1998) ecological model details.

The research value of longitudinal data is reinforced in assessing changes in children's development. But other research about specific development periods, including Blair et al.’s (2005) study of cortisol reactivity and executive function in 4- to 5-year-olds, is valuable to further knowledge. Furthermore, various data sources and approaches can complement efforts including “microlevel approaches, growth-curve modeling (e.g., van den Boom and Hoeksma, 1995), or qualitative data, to their correlational approach” (Paulussen-Hoogeboom et al., 2007, p.450) to advance the survival and development principle.

### Child participation

In all matters concerning him/her, CRC a.12 affirms the child's right to express his/her own views freely to be given “due weight,” but this principle remains a research challenge (Collins, 2007). While the research priority of mothers’ parenting role inspired a correct call for more attention to fathers (Paulussen-Hoogeboom et al., 2007), a CRBA demands meaningful child participation in studies about children. Children's own knowledge and ideas about issue(s) under consideration tend not to be studied (Grover, 2004), and yet offer an exciting new direction for psychological research. Children's contributions will inform understandings of, and approaches to children by others. While most children may not be aware of specific hormones or brain regions for instance, they can support research in many ways including identifying: research areas in neuroimaging and SES; and new emerging issues in SES. While not referring to child participation, Gianaros and Manuck (2010) have identified progress in neurobiological understanding of SES requires improvements in measurement and interpretation of SES indicators. They explain subjective SES measures “other than income, education, and occupation” could be used (Gianaros and Manuck, 2010, pp. 458–459). This future direction would benefit from children's contributions and advance research and understandings about children.

## Conclusion

In short, how do we approach and understand children? Science is not about what we know since it “is defined in terms of how and why we know something” (McCain and Segal, 1973, p. 36). CR are important in scientific inquiry in challenging assumptions, methods, tools, and results about children. A CRBA will complicate research efforts of SES impact upon children but will also improve research and understanding about children.

Brief recommendations to researchers to support a CRBA include:

- Improve CR understanding;
- Assess role and impact of CR upon research; and
- Incorporate a CRBA in research.

Researchers concerned about children should improve their CR knowledge and utilization since Eekelaar (1992, p. 234) explains:

“It would be a grievous mistake to see the Convention [CRC] applying to childhood alone.… The Convention is for all people. It could influence their entire lives.”
